# C-reactive Protein as a Predictor of Severity in Chronic Obstructive Pulmonary Disease: An Experience From a Tertiary Care Hospital

**DOI:** 10.7759/cureus.28229

**Published:** 2022-08-21

**Authors:** Afnan Hassan, Nosheen Jabbar

**Affiliations:** 1 Internal Medicine, Shifa International Hospital Islamabad, Islamabad, PAK; 2 General Medicine, Shifa International Hospital Islamabad, Islamabad, PAK

**Keywords:** pulmonary, severity, diagnostic, c-reactive protein, chronic obstructive pulmonary disease

## Abstract

Background

In this study, we aimed to determine the frequency of raised C-reactive protein (CRP) levels and their association with the severity of the disease.

Methodology

This descriptive cross-sectional study was conducted at the Shifa International Hospital, Islamabad, from June 2018 to December 2018 in the Department of Medicine. Patients attending the respiratory outpatient clinic in the Department of Medicine, Shifa International Hospital, Islamabad, with chronic obstructive pulmonary disease, meeting the sample selection criteria, were included in our study. A total of 104 patients were enrolled. All patients had plasma CRP levels measured, and forced expiratory volume in one second to forced vital capacity ratio was calculated to quantify the severity of the disease. We used SPSS version 26.0 (IBM Corp., Armonk, NY, USA) for data analysis.

Results

All patients with levels of hs-CRP greater than 3 mg/L had stage 3 or 4 chronic obstructive pulmonary disease (COPD) according to Global Initiative for Chronic Obstructive Lung Disease (GOLD) criteria, which accounted for 16.4% of the sample, while 81.7% of all patients suffering from COPD had levels greater than 1 mg/L. Only a small minority of patients, 1.9%, had normal high-sensitivity (hs)-CRP levels. The relationship between high levels of hs-CRP levels and advanced stages of COPD was statistically significant (p < 0.001).

Conclusions

The severity of COPD is directly related to the raised CRP levels, which can help in identifying these patients and managing them subsequently. It can be a useful indicator and a basis for high suspicion index and close follow-up for patients with high levels.

## Introduction

Chronic obstructive pulmonary disease (COPD) is currently the third most frequent cause of disability and mortality globally in patients older than 40, with an estimated prevalence of 174 million affected and 3.2 million mortalities worldwide in 2015, even though many studies postulate that these figures are likely understated [1,2]. The disorder is associated with preserved pulmonary symptoms brought on by airflow limitation secondary to respiratory airway damage caused by toxic gas or airborne particle exposure, which results in inflammation [3].

C-reactive protein (CRP) is a compound that increases in plasma during inflammatory processes. It helps to identify and remove infectious agents and damaged cells by binding to them, following which it activates the classic complement pathway and induces phagocytosis, ultimately helping in the killing mechanism of innate host defense [4]. Its levels wax and wane in direct proportion to the degree of inflammation, with persistently elevated levels seen in chronic pro-inflammatory disorders such as autoimmune diseases and chronic respiratory disorders [5].

In patients with acute exacerbations, CRP levels tend to increase, a conclusion reached after testing about 36 biomarkers, with good diagnostic accuracy, increased levels correlating with raised white blood cell count, and a purulent expectorate because of bacterial infection [6]. Studies have proposed that serial measurements of plasma CRP can lead to favorable outcomes of COPD when treated with inhaled corticosteroids and antibiotics [7,8]. Furthermore, CRP is also thought to be a contributing factor in determining the prognosis of COPD, as such increased levels are not only seen in acute exacerbations but also chronic, stable disease, indicating an ongoing low-level, underlying inflammatory activity, even when compared to smokers without the disease [9]. Elevated levels are also thought to be associated with decreased exercise tolerance due to disordered energy metabolism, increased cardiac disease, and all-cause mortality [10].

COPD is a significant source of disability and death. The disease is associated with acute and chronic disability, and the key to management lies in the early recognition and institution of treatment. CRP levels are proposed to be a possible marker for the severity of this disease, and may also play a role in the prediction of prognosis. This study was conducted to measure the association of raised CRP levels with disease severity in the Pakistani population.

## Materials and methods

We conducted this descriptive cross-sectional study from June 2018 to December 2018 in the outpatient clinic of the Department of Medicine at Shifa International Hospital, Islamabad. In total, 104 patients with the diagnosis of COPD were enrolled. Informed consent was taken, and patients were chosen via non-probability consecutive sampling. Ethical clearance was obtained from the relevant hospital authorities. The World Health Organization sample size calculator was used to calculate the sample size. To calculate our sample, the confidence level (1-ɑ) was kept at 96%; however, the anticipated population proportion (P) of 56.7% [11] and an absolute precision (d) of 0.1 was used. Patients aged 40-80 years with stable chronic COPD were included in the study. Patients who had recent exacerbations, ongoing infection, or received immunomodulatory drugs within the past month were excluded. Comorbidities such as autoimmune disease, bronchial asthma, respiratory malignancies, cardiovascular disease, or alpha-1-anti-trypsin deficiency were also taken into account, and any patient with these known diseases was excluded.

All patients were thoroughly evaluated by history and clinical examination on enrollment in the study in the outpatient clinic. All patients underwent a clean venepuncture performed by a registered phlebotomist with a minimum of 10 years of experience. A 3 mL blood sample was drawn and collected in a Gel Tube using a disposable syringe which underwent a quantitative analysis for high-sensitivity (hs) CRP levels in the chemical pathology lab. All patients also underwent spirometry for the measurement of lung volumes. Global Initiative for Chronic Obstructive Lung Disease (GOLD) system was used to stage COPD based on the ratio of forced expiratory volume in one second to forced vital capacity (FEV1/FVC). According to the GOLD classification, in patients with an FEV1/FVC ratio of less than 0.7, if FEV1 was more than or equal to 80%, it was stage 1 or mild COPD. If FEV1 was less than 50% of the predicted, COPD was staged 2, or moderate. For stages 3 and 4, the values for FEV1 were less than 30% of 50% predicted and less than 50%, respectively.

Data were analyzed using SPSS version 26.0 (IBM Corp., Armonk, NY, USA). Mean and standard deviation (SD) was calculated for quantitative variables such as age, body mass index (BMI), hs-CRP level, and the duration of illness. Gender and stage of disease were recorded regarding frequency and percentage as these were quantifiable. The chi-square test was applied to all variables for comparison with the severity of the disease. The association of hs-CRP levels with the COPD stage was measured using the one-way analysis of variance (ANOVA) test. P-values of ≤0.05 were considered significant.

## Results

Our sample included 104 patients, with a mean age of 63.54 ± 7.94 years. The majority of our population was male, 77 (74%), with a male-to-female ratio of 2.85:1. The mean duration of COPD in the complete sample was 3.84 ± 1.35 years. A total of 14 (13.5%) patients suffered from stage 1 COPD, 41 (39.4%) patients had stage 2 disease, while 34 (32.7%) and 15 (14.4%) patients suffered from stage 3 and 4 disease, respectively. The mean hs-CRP levels seen in our sample was 1.57 ± 1.99 mg/L. Results according to gender are shown in Table 1. Both advanced stage of disease and higher mean hs-CRP levels were associated with the male gender (p = 0.021 and p = 0.01, respectively).

**Table 1:** Results according to gender. hs-CRP: high-sensitivity C-reactive protein

Variable	Male	Female	P-value
Age (years)	63.49 ± 8.4	63.67 ± 6.5	0.923
Disease duration (years)	3.38 ± 1.23	5.15 ± 0.77	<0.001
Disease stage			
Stage 1	7 (9.1%)	7 (26.0%)	0.021
Stage 2	31 (40.3%)	10 (37.0%)	
Stage 3	24 (31.2%)	10 (37.0%)	
Stage 4	15 (19.4%)	0 (0%)	
hs-CRP levels (mg/L)	1.86 ± 2.25	0.73 ± 0.15	0.01

In addition, the advanced stages of COPD were associated with higher levels of hs-CRP (p < 0.001), as shown in Table 2.

**Table 2:** Association with COPD stage with hs-CRP levels. COPD: chronic obstructive pulmonary disease; hs-CRP: high-sensitivity C-reactive protein

COPD stage	hs-CRP level (mg/L)	P-value
Stage 1	0.61 ± 0.03	<0.001
Stage 2	0.73 ± 0.04	
Stage 3	0.95 ± 0.43	
Stage 4	6.16 ± 1.58	

## Discussion

The prognostic value of hs-CRP has already been proven in patients with ischemic heart disease [12]. However, its role in the surveillance of stable COPD is yet to be ascertained, especially regarding the association between raised CRP levels and increased mortality rates [13]. This is the subject of some controversy. Lomholt et al. conducted a meta-analysis on the subject and concluded that raised hs-CRP did not predict an increase in mortality in patients with COPD [13], which was refuted by Luezzi et als who proposed, in their meta-analysis, that there was an increased possibility of death showing a positive statistical relationship between raised CRP levels and increased mortality (hazard ratio (HR) = 1.53, 95% confidence interval (CI) = 1.32-1.77) [14].

Another controversial aspect is the cause of death in these patients. Pływaczewski et al. established that the majority of patients (48.8%) died from cardiovascular disease, while 23.3% died from respiratory failure, and 20.9% died from neoplastic disease in their study sample [15]. Anthonisen et al. reported that most of the patients in their study died from malignancies, 54%, while only 22% died from cardiovascular disease, and only a paltry 8% died from respiratory failure [16]. While the exact pathogenetic mechanism for cardiovascular mortality in COPD is yet to be ascertained, studies have shown that, in addition to raised hs-CRP, which has its own atherogenic properties [17], the levels of N-terminal pro-brain natriuretic peptide and troponin-T are raised despite normal ventricular function [18].

The pathogenic mechanism suggested for the relation between raised CRP levels and COPD is based on pulmonary inflammation. Prolonged smoke inhalation potentiates this process exponentially and persists at a low level even if the stimulus is ceased. Yi et al. conducted a systematic review of studies researching levels of CRP in children and adults on the persistent exposure to air pollution/particulate matter/smoke and found that there was an increase in CRP levels in such patients with prolonged exposure [19].

We based our cut-off value for CRP levels on the meta-analysis conducted by Luezzi et al., who postulated that a level greater than 3 mg/L may provide the best idea of a poorer outcome regarding late mortality [14]. Not only were these levels predictive for COPD concerning mortality but also for lung cancer and cardiovascular disease, which may be indicative of a common pathogenetic pathway culminating in death in all three diseases [20,21]. However, this issue requires further investigation as there is some evidence of causality, but the exact pathogenesis is yet to be determined clearly.

Because it is well established that there is systemic inflammation, the next question becomes: what to do about it? Anti-inflammatory drugs may be the answer. Statins are a group of drugs that have been increasingly recognized to have anti-inflammatory properties, having been demonstrated to reduce inflammation, as evidenced by the reduction in mean CRP levels [22]. A meta-analysis conducted by Lu et al. in 2019 demonstrated that multiple HMG-CoA reductase inhibitors (statins) decreased the risk of all-cause mortality, cardiovascular disease-related mortality, and mortality due to respiratory failure in patients suffering from COPD patients, with the relative risk (RR) (95% CI) of 0.72 (0.63, 0.84), 0.72 (0.53, 0.98), and 0.84 (0.79, 0.89), respectively, and definitively showed that reduced CRP levels significantly [23].

Here we would like to add that Lahousse et al. demonstrated the use of prolonged statin therapy in patients with COPD and found a 78% reduction in all-cause mortality in cases where the baseline level of hs-CRP was greater than 3 mg/L (our cut-off value), while the benefit in mortality was a modest 21% if the baseline CRP level was less than 3 mg/L [24]. Thus, this level is not only useful as a cut-off for the classification of mortality risk in these patients but may also serve as an indicator for the initiation of immunomodulatory drugs such as statins.

Other drugs used for immune modulation in cases with COPD include macrolides and quinolones; both classes are known to have immune-modifying effects in addition to their antibacterial effects, which may be beneficial in COPD, especially in acute exacerbations [25]. Vitamin D, which has purported immunomodulatory effects, is thought to be helpful in pulmonary infections such as coronavirus disease 2019 and has also been proposed to be beneficial in COPD [26]. At the same time, aspirin has also been useful [27]. Less conventional therapies include laser therapy and acupuncture to increase exercise tolerance and the strength of the muscles of respiration, which also have immunomodulatory side effects. However, most of these therapeutic interventions need further research before being employed regularly.

Lastly, the association of raised CRP levels with acute exacerbation of COPD is well established, but its role as a prognostic marker in such cases is less so. Gallego et al. proposed that raised CRP in acute exacerbation is associated with certain types of bacterial pneumonia complicating COPD and predictive of increased requirements for hospital admission, intensive care, and more extended in-hospital stay. However, Leuzzi et al. disagreed with these findings and demonstrated that the relationship between raised CRP and early mortality was relatively weak [14]. As there is some conflict on the issue, we propose that further investigation is necessary to clarify this association.

This study was conducted only at one center, which was limited by the lack of long-term follow-up. In addition, our measurements were based on spot-checks, with little monitoring of each patient over the long term to identify rhythms and trends, which may be seen in diseases that wax and wane, such as COPD.

## Conclusions

This research demonstrates that patients with stable COPD have raised levels of serum CRP, which correlates with the severity of COPD and may also correlate with the occurrence of complications, indicating a possible role in the surveillance and management of such patients. The disorder results in systemic inflammation, which results in the progression of the disease, morbidity, and mortality. It must be pointed out that raised CRP levels are associated with multiple pathological processes associated with COPD, and as such further research is required to understand its therapeutic and prognostic role.

## References

[1] Singh D, Agusti A, Anzueto A (2019). Global strategy for the diagnosis, management, and prevention of chronic obstructive lung disease: the GOLD science committee report 2019. Eur Respir J.

[2] (2017). Global, regional, and national deaths, prevalence, disability-adjusted life years, and years lived with disability for chronic obstructive pulmonary disease and asthma, 1990-2015: a systematic analysis for the Global Burden of Disease Study 2015. Lancet Respir Med.

[3] (2022). Global Strategy for the Diagnosis, management and prevention of COPD, Global Initiative for Chronic Obstructive Lung Disease. https://goldcopd.org/wp-content/uploads/2018/11/GOLD-2019-v1.7-FINAL-14Nov2018-WMS.pdf.

[4] Jungen MJ, Ter Meulen BC, van Osch T, Weinstein HC, Ostelo RW (2019). Inflammatory biomarkers in patients with sciatica: a systematic review. BMC Musculoskelet Disord.

[5] Nehring SM, Goyal A, Patel BC (2022). C Reactive Protein. https://www.ncbi.nlm.nih.gov/books/NBK441843/.

[6] Francis NA, Gillespie D, White P (2020). C-reactive protein point-of-care testing for safely reducing antibiotics for acute exacerbations of chronic obstructive pulmonary disease: the PACE RCT. Health Technol Assess.

[7] Oshagbemi OA, Franssen FM, Wouters EF, Maitland-van der Zee AH, Driessen JH, de Boer A, de Vries F (2020). C-reactive protein as a biomarker of response to inhaled corticosteroids among patients with COPD. Pulm Pharmacol Ther.

[8] An X, Zhang C, Weng X, Xiao W, Sun Z, Zeng Z, Huang Q (2020). C-reactive protein testing to guide antibiotic prescribing for COPD exacerbations: a protocol for systematic review and meta-analysis. Medicine (Baltimore).

[9] Aksu F, Capan N, Aksu K, Ofluoğlu R, Canbakan S, Yavuz B, Akin KO (2013). C-reactive protein levels are raised in stable chronic obstructive pulmonary disease patients independent of smoking behavior and biomass exposure. J Thorac Dis.

[10] Corbo GM, Inchingolo R, Sgueglia GA, Lanza G, Valente S (2013). C-reactive protein, lung hyperinflation and heart rate variability in chronic obstructive pulmonary disease --a pilot study. COPD.

[11] Polyakova EA, Mikhaylov EN (2020). The prognostic role of high-sensitivity C-reactive protein in patients with acute myocardial infarction. J Geriatr Cardiol.

[12] Garcia-Rio F, Miravitlles M, Soriano JB (2010). Systemic inflammation in chronic obstructive pulmonary disease: a population-based study. Respir Res.

[13] Lomholt FK, Laulund AS, Bjarnason NH, Jørgensen HL, Godtfredsen NS (2014). Meta-analysis of routine blood tests as predictors of mortality in COPD. Eur Clin Respir J.

[14] Leuzzi G, Galeone C, Taverna F, Suatoni P, Morelli D, Pastorino U (2017). C-reactive protein level predicts mortality in COPD: a systematic review and meta-analysis. Eur Respir Rev.

[15] Pływaczewski R, Maciejewski J, Bednarek M, Zieliński J, Górecka D, Śliwiński P (2015). Causes of deaths in COPD patients in primary care setting--a 6-year follow-up. Pneumonol Alergol Pol.

[16] Anthonisen NR, Skeans MA, Wise RA, Manfreda J, Kanner RE, Connett JE (2005). The effects of a smoking cessation intervention on 14.5-year mortality: a randomized clinical trial. Ann Intern Med.

[17] Badimon L, Peña E, Arderiu G, Padró T, Slevin M, Vilahur G, Chiva-Blanch G (2018). C-reactive protein in atherothrombosis and angiogenesis. Front Immunol.

[18] Adrish M, Nannaka VB, Cano EJ, Bajantri B, Diaz-Fuentes G (2017). Significance of NT-pro-BNP in acute exacerbation of COPD patients without underlying left ventricular dysfunction. Int J Chron Obstruct Pulmon Dis.

[19] Li Y, Rittenhouse-Olson K, Scheider WL, Mu L (2012). Effect of particulate matter air pollution on C-reactive protein: a review of epidemiologic studies. Rev Environ Health.

[20] Fonseca FA, Izar MC (2016). High-sensitivity C-reactive protein and cardiovascular disease across countries and ethnicities. Clinics (Sao Paulo).

[21] Allin KH, Nordestgaard BG (2011). Elevated C-reactive protein in the diagnosis, prognosis, and cause of cancer. Crit Rev Clin Lab Sci.

[22] Soedamah-Muthu SS, Livingstone SJ, Charlton-Menys V (2015). Effect of atorvastatin on C-reactive protein and benefits for cardiovascular disease in patients with type 2 diabetes: analyses from the Collaborative Atorvastatin Diabetes Trial. Diabetologia.

[23] Lu Y, Chang R, Yao J, Xu X, Teng Y, Cheng N (2019). Effectiveness of long-term using statins in COPD - a network meta-analysis. Respir Res.

[24] Lahousse L, Loth DW, Joos GF, Hofman A, Leufkens HG, Brusselle GG, Stricker BH (2013). Statins, systemic inflammation and risk of death in COPD: the Rotterdam study. Pulm Pharmacol Ther.

[25] Blasi F, Mantero M, Aliberti S (2012). Antibiotics as immunomodulant agents in COPD. Curr Opin Pharmacol.

[26] Heulens N, Korf H, Janssens W (2015). Innate immune modulation in chronic obstructive pulmonary disease: moving closer toward vitamin D therapy. J Pharmacol Exp Ther.

[27] Fawzy A, Putcha N, Aaron CP (2019). Aspirin use and respiratory morbidity in COPD: a propensity score-matched analysis in subpopulations and intermediate outcome measures in COPD study. Chest.

